# Attitude, Awareness and Knowledge of Telemedicine Among Romanian Medical Students: A Cross‐Sectional Study

**DOI:** 10.1002/hsr2.72695

**Published:** 2026-06-22

**Authors:** Andreea‐Ramona Treteanu, Ștefan Busnatu, Liviu‐Ionuț Șerbănoiu, Iulian Năstasă, Octavian Andronic

**Affiliations:** ^1^ Carol Davila University of Medicine and Pharmacy Bucharest Romania; ^2^ Innovation and e‐Health Center Carol Davila University of Medicine and Pharmacy Bucharest Romania; ^3^ Bagdasar‐Arseni Clinical Emergency Hospital Bucharest Romania

**Keywords:** digital health, health education, medical students, Romania, telemedicine

## Abstract

**Background and Aims:**

Telemedicine has become an essential element of contemporary healthcare, offering new ways to connect patients and clinicians, improve access to medical services and streamline clinical workflows. Although the COVID‐19 pandemic accelerated its worldwide adoption, successful integration still relies on healthcare professionals' openness, preparedness and confidence in using these technologies. In Romania, evidence regarding medical students' knowledge and attitudes toward telemedicine remains limited. This study aimed to assess Romanian medical students' awareness, attitudes and perceived barriers regarding telemedicine and to evaluate their preparedness for integrating digital health technologies into future clinical practice.

**Methods:**

A nationwide cross‐sectional survey was conducted between 1 October and 15 November 2024 among medical students from all Romanian university centers. Data were collected via a structured online questionnaire (21 items) addressing demographics, knowledge, experiences and perceptions of telemedicine. Descriptive and inferential statistics were used to analyze associations between demographic factors, familiarity and willingness to adopt telemedicine.

**Results:**

A total of 608 students participated. 61.7% were familiar with telemedicine, yet only 11.7% had used it personally. Most respondents (78.8%) believed telemedicine improves healthcare quality and 84.4% supported its inclusion in medical education, although 86% had not received formal training. Reported advantages included time efficiency (81%) and improved access to specialists (76%); the main drawbacks were limited physical assessment (88.7%) and reduced interpersonal interaction (74.7%). Younger students showed significantly higher familiarity (*p* < 0.001).

**Conclusions:**

Romanian medical students demonstrate positive attitudes toward telemedicine but lack structured training and practical exposure. Integrating telemedicine into medical curricula and improving digital infrastructure are essential to prepare future physicians for technology‐enabled healthcare delivery.

## Summary

**What Is Known:**

- Telemedicine enhances healthcare access and efficiency but requires adequate professional readiness for effective implementation.

**What Is New:**

- This is the first nationwide study exploring Romanian medical students' knowledge, attitudes and perceptions toward telemedicine.

**Clinical Implications:**

- Integrating telemedicine education into medical curricula and addressing infrastructural barriers are vital steps toward sustainable digital healthcare integration in Romania.

## 1 Introduction

Telemedicine, broadly defined as the remote diagnosis, treatment and monitoring of patients through telecommunications technology, has emerged as a key component of modern healthcare [1]. The integration of digital tools, including video consultations, mobile health applications and telemonitoring systems, has transformed the delivery of medical services by expanding access to care and enabling more flexible models of healthcare delivery. Its rapid expansion, particularly during the COVID‐19 pandemic, has accelerated adoption worldwide across both developed and developing countries [2].

At the global level, telemedicine has been increasingly incorporated into healthcare systems as part of the broader digital transformation of medicine. Within this context, Romania has also made progress in this field, with early initiatives dating back to the early 2000s, when pilot projects aimed to establish a national telemedicine network [3]. Although these initial efforts were hindered by technological and regulatory limitations, the legislative framework has been significantly strengthened in recent years through measures such as Government Emergency Ordinance No. 196/2020. These developments have enabled the implementation of services such as teleconsultation, telemonitoring and teleradiology, reflecting growing institutional support for digital healthcare solutions.

The successful integration of telemedicine into routine practice depends not only on infrastructure and policy, but also on the readiness and acceptance of healthcare professionals. Previous research conducted among Romanian specialists has highlighted the role of professional attitudes in shaping telemedicine adoption [4]. In this context, understanding the perspectives of medical students, as future healthcare providers, is essential.

Across the world, previous research investigating medical students' perceptions of telemedicine has consistently reported generally positive attitudes toward its implementation, alongside concerns related to data privacy, ethical issues and insufficient training within medical curricula [5, 6, 7]. These findings underscore the importance of evaluating students' preparedness to engage with digital health technologies.

However, evidence on this topic in Romania remains limited. A recent study explored Romanian medical students' perceptions of telemedicine integrated with artificial intelligence [8], but comprehensive data focusing specifically on telemedicine as an independent concept are lacking. This research gap highlights the necessity of further investigation to understand their readiness, perceived barriers and educational needs in relation to telemedicine.

This article aims to explore the perceptions of medical students in Romania regarding the use of telemedicine for optimizing medical activities. By assessing their knowledge, attitudes and perceived barriers, this study seeks to provide valuable insights into their readiness to embrace digital health technologies and contribute to the ongoing development of telemedicine in Romania. The results are intended to inform targeted educational initiatives and policy recommendations to support the seamless integration of telemedicine into the Romanian healthcare landscape.

## 2 Materials and Method

### 2.1 Study Design and Reporting Guidelines

This study was designed as an observational, descriptive, cross‐sectional survey conducted using an online questionnaire developed on the Google Forms platform. The reporting of this study was aligned with the Strengthening the Reporting of Observational Studies in Epidemiology (STROBE) guidelines for cross‐sectional studies and the Checklist for Reporting Results of Internet E‐Surveys (CHERRIES). A completed STROBE checklist is provided as supporting material.

### 2.2 Study Setting and Period

Data collection was carried out between 1 October and 15 November 2024.

### 2.3 Study Population, Sampling and Eligibility Criteria

The target population consisted of medical students enrolled in Romanian medical universities across all university centers. Students were eligible to participate regardless of year of study or demographic characteristics, provided that they were currently enrolled in a Romanian medical university and agreed to participate by providing informed consent. Responses were excluded if questionnaires were substantially incomplete (> 20% missing responses) or identified as potential duplicates. A non‐probability convenience sampling strategy was used.

### 2.4 Sample Size Consideration

No a priori sample size calculation was performed due to the exploratory nature of the study. However, the final sample size of 608 participants exceeds the minimum recommended sample size for cross‐sectional surveys estimating proportions with a 95% confidence level and a 5% margin of error, assuming a conservative response distribution of 50%, which would require approximately 384 participants. Therefore, the achieved sample size was considered adequate to ensure sufficient statistical power.

### 2.5 Questionnaire Design

The questionnaire was adapted from instruments previously described in the published literature [9, 10, 11, 12]. The adaptation process involved a review of relevant studies, followed by modification of items to reflect the Romanian educational and healthcare context. Content and face validity were assessed through evaluation by two academic experts with experience in telemedicine and medical education. Subsequently, the questionnaire was pilot tested on a group of 20 medical students to assess clarity, comprehensibility and completion time. Based on the feedback obtained, revisions were made to improve wording and structure.

The final questionnaire comprised 21 items organized into three main sections addressing demographic characteristics, knowledge and experience with telemedicine, and perceptions regarding its advantages, disadvantages and future use in medical practice. The full version of the questionnaire is presented in Table 1.

**Table 1:** Items of the research questionnaire.

**Informed consent**
**Demographic data:**
1. What is your age?
2. What is your gender?
3. What year of study are you currently in?
4. At which university center are you studying?
**Knowledge and experiences with telemedicine**
5. Are you familiar with the concept of telemedicine?
6. If yes, how would you describe telemedicine in your own words?
7. Have you ever used telemedicine services as a patient?
8. If yes, what type of service did you request?
9. To what extent are you familiar with recent innovations in medicine?
10. What are your sources of information about telemedicine?
**Perceptions of telemedicine's advantages and disadvantages**
11. What do you consider to be the main advantages of telemedicine?
12. What do you consider to be the main disadvantages of telemedicine?
**Perceptions and professional training in telemedicine**
13. For which of the following types of medical services do you think telemedicine can be used?
14. Do you believe telemedicine would positively or negatively influence the quality of medical services?
15. To what extent do you agree with the following statements about telemedicine?
16. Have you participated in or are you currently benefiting from training programs related to the use of telemedicine? If yes, in what format?
17. Do you find training programs on telemedicine usage useful for your education as future healthcare professionals?
18. What is your opinion on introducing courses about telemedicine into the university curriculum of medical faculties?
19. To what extent do you believe telemedicine can contribute to optimizing medical activities?
**Perceptions regarding the use of telemedicine in your future professional career**
20.To what extent are you willing to include telemedicine in your practice to provide patient care?
21.What improvements do you think could be made to telemedicine services to make them more effective?

Considering that the questionnaire was composed predominantly of single‐item measures addressing conceptually distinct domains, the assessment of internal consistency reliability and the application of factor analysis were not deemed methodologically appropriate. Accordingly, each item was analyzed independently, in line with the exploratory nature of the study.

### 2.6 Data Collection Procedure

The questionnaire was distributed exclusively online, through social media groups dedicated to medical students and via institutional email channels. Two reminder messages were sent at approximately 1‐month intervals to increase the response rate. Measures implemented to ensure data quality included: restriction to one response per Google account, screening for duplicate entries during data cleaning, exclusion of incomplete questionnaires. The total number of students invited could not be precisely determined due to the distribution method; therefore, a response rate could not be calculated. A total of 615 responses were initially collected. After applying the predefined exclusion criteria, 7 questionnaires were excluded due to substantial incompleteness (> 20% missing data). The final sample consisted of 608 participants included in the analysis.

### 2.7 Statistical Analysis

Data were exported from Google Forms and analyzed using JASP version 0.96.0.0. Descriptive statistics, including means, standard deviations, frequencies and percentages, were used to summarize the data. Categorical variables were summarized as frequencies and percentages, while continuous variables were expressed as means and standard deviations. Associations between categorical variables were assessed using the chi‐square test.

Multivariable logistic regression was performed for binary outcomes, while ordinal logistic regression was used for Likert‐scale outcomes. Results are presented as odds ratios (ORs) with 95% confidence intervals (CIs). All multivariable models were adjusted for age, gender, year of study, prior telemedicine training, and familiarity with recent medical innovations.

Statistical significance was defined as a two‐sided *p*‐value < 0.05. Missing data were handled by excluding incomplete questionnaires based on predefined criteria. Age was treated as a continuous variable in the regression analyses.

Open‐ended responses regarding the definition of telemedicine were analyzed using a qualitative content analysis approach. A response was considered correct if it included key conceptual elements, such as the remote provision of healthcare services and the use of information and communication technologies. The coding of responses was performed independently by two authors and any discrepancies were resolved through discussion until consensus was reached.

### 2.8 Ethical Considerations

Ethical approval for the study was obtained from the Research Ethics Committee of the “Carol Davila” University of Medicine and Pharmacy in Bucharest (approval no. 8364/29.03.2024). The study was conducted in accordance with the ethical principles outlined in the Declaration of Helsinki and relevant national regulations. Prior to completing the questionnaire, all participants were informed about the purpose of the study and provided informed consent electronically. Participation was voluntary, and confidentiality of the collected data was strictly maintained.

## 3 Results

### 3.1 Demographic Characteristics

A total of 608 medical students completed the questionnaire. The demographic characteristics of the study group are summarized in Table 2. The mean age was 21.36 years (SD = 2.67), with a median of 21 years (range: 18–44).

**Table 2:** Demographic characteristics of the study group.

Age	(years)
Mean	21.359
Median	21
SD	2.67
Min	18
Max	44

Gender	No. (%)
Female	485 (79.7%)
Male	114 (18.8%)
Students who did not disclose their gender	9 (1.5%)

Year at medical school	No. (%)
First year	183 (30.1%)
Second year	98 (16.1%)
Third year	91 (15%)
Fourth year	135 (22.2%)
Fifth year	55 (9%)
Sixth year	46 (7.6%)

Most respondents were female (79.7%, *n* = 485), followed by male participants (18.8%, *n* = 114). A small proportion of respondents (9 students, 1.5%) chose not to disclose their gender.

First‐year students represented the largest group (30.1%, *n* = 183), followed by fourth‐year (22.2%, *n* = 135) and second‐year students (16.1%, *n* = 98).

### 3.2 Knowledge and Experience with Telemedicine

A total of 61.7% (*n* = 375) of respondents reported being familiar with the concept of telemedicine, while 38.3% (*n* = 233) indicated a lack of familiarity (Table 3). Among those familiar, most were able to provide a correct definition of the term. Only 11.7% (*n* = 71) of participants had previously used telemedicine services as patients, whereas 88.3% (*n* = 537) reported no prior use.

**Table 3:** Perceptions, knowledge and experiences with telemedicine.

Item	Frequency (%)	
**Are you familiar with the concept of telemedicine?**		
Yes	375 (61.7%)	
No	233 (38.3%)	
**Have you ever used telemedicine services as a patient?**		
Yes	71 (11.7%)	
No	537 (88.3%)	
**What are your sources of information about telemedicine?**	Yes	No
University courses	267 (43.9%)	341 (56.1%)
Internet	530 (87.2%)	78 (12.8%)
Colleagues/Friends	358 (58.9%)	250 (41.1%)
Medical practice	203 (33.4%)	405 (66.6%)
**Do you consider that telemedicine would positively or negatively influence the quality of healthcare services?**		
Positively	479 (78.8%)	
Negatively	129 (21.2%)	
**Have you benefited from training programs regarding the use of telemedicine?**		
No	523 (86%)	
Yes, at university	34 (5.6%)	
Yes, self‐education	62 (10.2%)	
**Do you find training programs on the use of telemedicine useful in your formation as future health professionals?**		
Yes	513 (84.4%)	
No	95 (15.6%)	

Reported uses of telemedicine included family medicine and dermatology consultations, reevaluation, interpretation of medical investigations, prescription issuance and postoperative follow‐up.

The internet was the most frequently reported source of information (87.2%, *n* = 530), followed by colleagues or friends (58.9%, *n* = 358) and university courses (43.9%, *n* = 267). Medical practice was less frequently reported (33.4%, *n* = 203) (Table 3).

Most respondents (86%, *n* = 523) reported no prior training in telemedicine. Training through university courses was reported by 5.6% (*n* = 34), while 10.2% (*n* = 62) indicated self‐directed learning.

A total of 78.8% (*n* = 479) of respondents perceived telemedicine as having a positive impact on healthcare quality, while 21.2% (*n* = 129) reported a negative perception. The majority (84.4%, *n* = 513) considered telemedicine training programs beneficial for their development as future healthcare professionals.

### 3.3 Advantages, Disadvantages and Future Use of Telemedicine in Medical Practice

**Figure 1:**
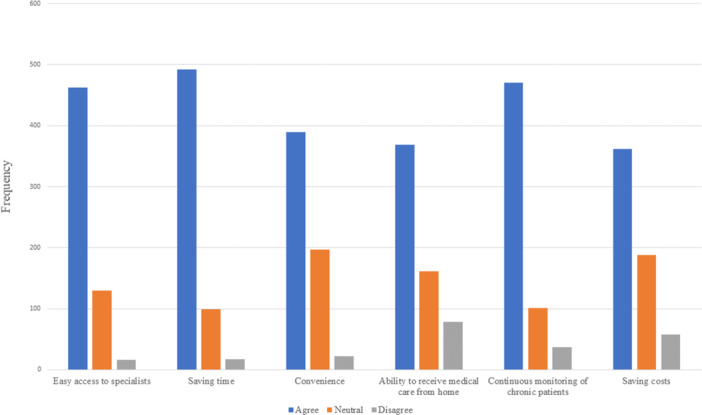
Advantages of telemedicine.

**Figure 2:**
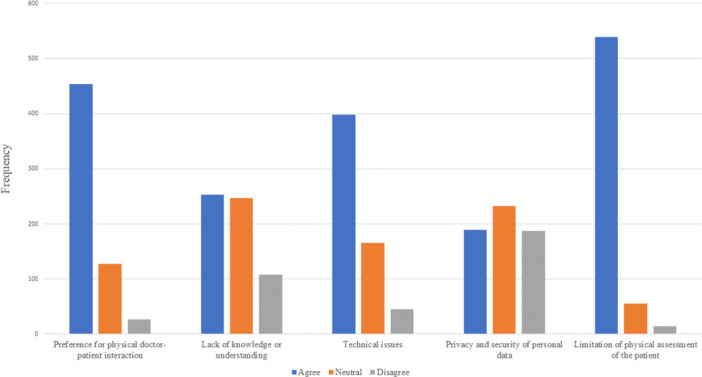
Disadvantages of telemedicine.

The perceived advantages and disadvantages of telemedicine are presented in Figure 1 and Figure 2.

The most frequently reported advantages were time savings (81%, *n* = 492), continuous monitoring of patients with chronic conditions (77.3%, *n* = 470), improved access to specialists (76%, *n* = 462) and the convenience of remote medical care (64%, *n* = 389). Cost savings and receiving care from home were also commonly reported.

The main reported disadvantages included the inability to perform physical examination (88.7%, *n* = 539), preference for face‐to‐face interaction (74.7%, *n* = 454) and technical issues (65.5%, *n* = 398). Other concerns included limited knowledge about telemedicine (41.6%, *n* = 253) and issues related to privacy and data security (31.1%, *n* = 189).

**Figure 3:**
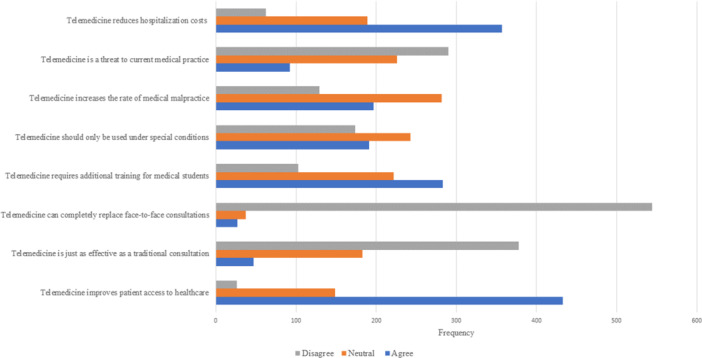
Respondents' perception of telemedicine.

Responses to statements regarding telemedicine are presented in Figure 3. A total of 71.2% (*n* = 433) of respondents agreed that telemedicine improves patient access to healthcare. In contrast, 7.7% (*n* = 47) considered telemedicine as effective as traditional consultations, while 77.4% (*n* = 378) disagreed.

Only 4.4% (*n* = 27) of respondents agreed that telemedicine could replace face‐to‐face consultations, whereas 89.3% (*n* = 544) disagreed. Regarding the need for additional training, 46.5% (*n* = 283) agreed, 36.5% (*n* = 222) reported a neutral position, and 17% (*n* = 103) disagreed. Additionally, 58.7% (*n* = 357) considered that telemedicine may reduce hospitalization costs, while 31.1% (*n* = 189) were neutral and 10.2% (*n* = 62) disagreed.

For the statement that telemedicine should be used only in special circumstances, 31.4% (*n* = 191) agreed, 40% (*n* = 243) were neutral and the remainder disagreed. A total of 32.4% (*n* = 197) of respondents agreed that telemedicine increases the risk of medical malpractice, while 15.1% (*n* = 92) considered it a threat to current medical practice.

**Figure 4:**
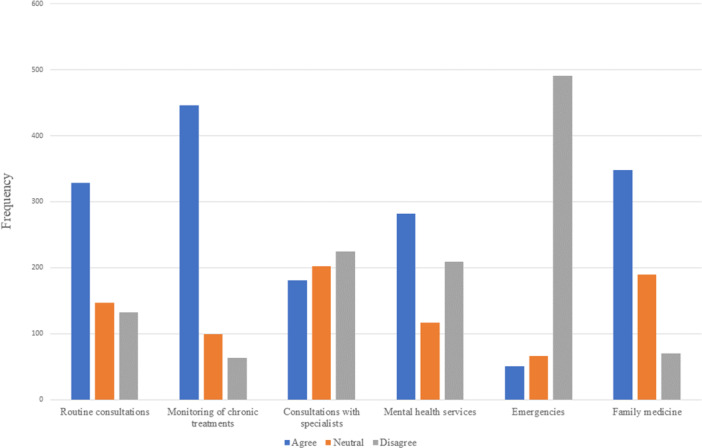
Respondents' perceptions of the utility of telemedicine services across different medical contexts.

Perceptions of the utility of telemedicine across different medical contexts are presented in Figure 4. Agreement was highest for monitoring of chronic treatments (*n* = 446) and for family medicine and routine consultations (*n* = 348 and *n* = 329, respectively). More heterogeneous responses were observed for specialist consultations and mental health services, with a substantial proportion of neutral responses (*n* = 202 and *n* = 117, respectively). Telemedicine was least supported for emergency care, with 491 respondents expressing disagreement.

Regarding the integration of telemedicine courses into the university curriculum, most respondents considered this initiative beneficial. Suggested formats included optional courses, workshops, or mandatory courses without a final grade.

Perceptions regarding the role of telemedicine in optimizing medical activities are presented in Table 4. A total of 46.5% of respondents expressed agreement (14.6% strongly agree, n = 89; 31.9% agree, n = 194), while 14.3% expressed disagreement (10% disagree, n = 61; 4.3% strongly disagree, n = 26). A neutral response was reported by 39.1% of participants.

**Table 4:** Medical students' perceptions of telemedicine's contribution to medical practice optimization and its integration into clinical care.

	Level of response, *n* (%)				
To what extent do you consider that telemedicine can contribute to the optimization of medical practice?	Strongly agree	Agree	Neutral	Disagree	Strongly disagree
	89 (14.6%)	194 (31.9%)	238 (39.1%)	61 (10%)	26 (4.3%)
To what extent are you willing to include telemedicine in your practice to provide patient care?	Strongly agree	Agree	Neutral	Disagree	Strongly disagree
	140 (23%)	173 (28.5%)	190 (31.3%)	79 (13%)	26 (4.3%)

Regarding willingness to adopt telemedicine in future clinical practice, 51.5% of respondents expressed agreement (140 strongly agree, 173 agree). Conversely, 17.3% (79 disagree, 26 strongly disagree) opposed its integration, while 31.3% maintained a neutral position.

### 3.4 Attitudes Toward Telemedicine and Analysis of Neutral Responses

Responses to attitudinal statements are presented in Figure 3. Neutral responses were analyzed as a distinct category across all items. Their proportion ranged from 6.1% to 46.4%, depending on the statement.

The highest proportions of neutral responses were observed for the perceived risk of medical malpractice (46.4%), the use of telemedicine only in special circumstances (40%), telemedicine as a threat to current medical practice (37.2%), the need for additional training (36.5%), and the potential to reduce hospitalization costs (31.1%). Lower proportions were observed for the replacement of face‐to‐face consultations (6.1%).

Neutral responses were also substantial for the perceived role of telemedicine in optimizing medical activities (39.1%) and for willingness to adopt telemedicine in future clinical practice (31.3%).

Additional analyses examined neutral responses according to familiarity with telemedicine. Across all attitudinal items, higher proportions of neutral responses were observed among respondents who were not familiar with telemedicine compared to those who were familiar.

Neutral responses for telemedicine improving access to healthcare were reported by 33.5% of non‐familiar respondents compared to 18.9% of familiar respondents. For perceived effectiveness compared to traditional consultations, the values were 33.0% versus 28.3%, respectively. For the replacement of face‐to‐face consultations, neutral responses were 9.4% among non‐familiar respondents and 4.0% among familiar respondents.

For the need for additional training, neutral responses were 39.1% among non‐familiar respondents compared to 34.9% among familiar respondents. Neutral responses for the use of telemedicine only in special circumstances were similar between groups (40.3% vs 39.7%), while for reduction of hospitalization costs they were 33.5% and 29.6%, respectively.

The highest proportions of neutral responses in both groups were observed for perceived malpractice risk (50.6% vs 43.7%) and telemedicine as a potential threat to current medical practice (45.1% vs 32.3%).

### 3.5 Factors Associated With Telemedicine‐Related Outcomes

Bivariate analysis (Table 5) identified a significant association between age and familiarity with telemedicine (*p* < 0.001). No significant association was observed between gender and the use of telemedicine services (*p* = 0.226). Academic year was associated with familiarity with recent medical innovations (*p* = 0.004). Familiarity with telemedicine was associated with its use (*p* < 0.001).

**Table 5:** Bivariate associations between key variables.

Variable 1	Variable 2	Test used	*p* value
Age	Familiarity with telemedicine	Chi‐square	< 0.001
Gender	Use of telemedicine	Chi‐square	0.226
Academic year	Familiarity with innovations	Chi‐square	0.004
Familiarity with telemedicine	Use of telemedicine	Chi‐square	< 0.001
Training	Perceived utility	Chi‐square	0.773
Training	Willingness to adopt	Chi‐square	0.333
Familiarity with innovations	Specialist consultations	Chi‐square	< 0.001
Telemedicine use	Perceived quality	Chi‐square	0.061

No significant associations were identified between prior telemedicine training and perceived utility (*p* = 0.773), nor between prior training and students' willingness to adopt telemedicine (*p* = 0.333). A significant association was observed between familiarity with medical innovations and the perceived potential of telemedicine in specialist consultations (*p* < 0.001). The association between telemedicine use and perceived impact on healthcare quality did not reach statistical significance (*p* = 0.061).

To further explore these relationships, multivariable regression analyses were performed, adjusting for age, gender, year of study, prior telemedicine training, and familiarity with recent medical innovations.

In the logistic regression model (Table 6), familiarity with telemedicine was independently associated with year of study (OR = 1.309, 95% CI: 1.129–1.516, *p* < 0.001) and familiarity with recent medical innovations (OR = 1.653, 95% CI: 1.357–2.014, *p* < 0.001). Age, gender, and prior telemedicine training were not significant predictors after adjustment (all *p* > 0.05).

**Table 6:** Logistic regression models for binary outcomes.

Variable	Familiarity with telemedicine OR (95% CI)	*p*‐value	Positive perception OR (95% CI)	*p* value
Age	—	0.367	—	0.729
Gender	—	0.957	—	0.763
Year of study	1.309 (1.129–1.516)	< 0.001	—	0.374
Training	—	0.756	—	0.398
Familiarity with innovations	1.653 (1.357–2.014)	< 0.001	1.393 (1.123–1.728)	0.003

*Note:* Models adjusted for age, gender, year of study, prior telemedicine training, and familiarity with recent medical innovations.

Abbreviations: CI, confidence interval; OR, odds ratio; “‐“, not statistically significant.

Perceived positive impact of telemedicine on healthcare quality was independently associated only with familiarity with recent medical innovations (OR = 1.393, 95% CI: 1.123–1.728, *p* = 0.003), while no significant associations were observed for age, gender, year of study, or prior training.

Ordinal logistic regression (Table 7) identified familiarity with recent medical innovations (OR = 1.83, 95% CI: 1.55–2.16, *p* < 0.001) and prior telemedicine training (OR = 1.78, 95% CI: 1.15–2.75, *p* = 0.010) as independent predictors of higher perceived contribution of telemedicine to medical activity optimization. Age, gender, and year of study were not significantly associated with this outcome.

**Table 7:** Ordinal logistic regression models for Likert‐scale outcomes.

Variable	Optimization OR (95% CI)	*p* value	Willingness OR (95% CI)	*p* value
Age	—	0.924	—	0.346
Gender	—	0.097	1.74 (1.20–2.52)	0.003
Year of study	—	0.667	—	0.391
Training	1.78 (1.15–2.75)	0.010	—	0.104
Familiarity with innovations	1.83 (1.55–2.16)	< 0.001	1.61 (1.37–1.89)	< 0.001

*Note:* Models adjusted for age, gender, year of study, prior telemedicine training, and familiarity with recent medical innovations.

Abbreviations: CI, confidence interval; OR, odds ratio; “‐“, not statistically significant.

Willingness to incorporate telemedicine into future clinical practice was independently associated with gender (OR = 1.74, 95% CI: 1.20–2.52, *p* = 0.003) and familiarity with recent medical innovations (OR = 1.61, 95% CI: 1.37–1.89, *p* < 0.001). No significant associations were identified for age, year of study, or prior telemedicine training.

### 3.6 Qualitative Feedback

At the end of the survey, respondents provided suggestions for improving telemedicine services. Frequently reported themes included increasing internet accessibility, improving technological infrastructure, enhancing data security, increasing public awareness and implementing centralized digital systems for data management.

## 4 Discussions

The COVID‐19 pandemic has brought telemedicine to the forefront of medical practice, highlighting its potential to optimize healthcare delivery, particularly in situations where face‐to‐face interactions are limited. This study aimed to explore the perceptions of medical students in Romania regarding the use of telemedicine to enhance medical activities, offering insights into the next generation of healthcare professionals' readiness to integrate digital tools into their practice.

The demographic profile of the study group, with a majority of respondents being female (79.7%), aligns with findings in previous research by Woloschuk et al., which suggests that women in healthcare may show greater interest in telemedicine [13]. This trend reflects broader gender‐based differences in engagement with medical technology, although further exploration of underlying factors is warranted. Additionally, the notable participation from first‐ and fourth‐year students may reflect different levels of academic and clinical exposure, with first‐year students being keenly interested in new medical concepts and fourth‐year students possessing sufficient clinical experience to contextualize telemedicine.

A significant finding is that 61.7% of respondents reported familiarity with telemedicine and most demonstrated an accurate understanding of its definition. This suggests a foundational awareness among students, likely driven by increased exposure during the pandemic. However, only 11.7% had personal experience using telemedicine services, indicating that despite awareness, practical engagement remains limited. This lack of personal use might reflect the nascent stage of telemedicine adoption in Romania.

The internet emerged as the principal source of information about telemedicine for 87.2% of respondents, consistent with findings by AlGhamdi & Moussa [14], underscoring its role as a critical resource for medical students. University courses (43.9%) and peer interactions (58.9%) also contributed significantly, while medical practice was less frequently cited (33.4%). These trends suggest the need for more structured and practical telemedicine training within formal curricula to complement independent learning.

Most students (78.8%) believed that telemedicine positively impacts healthcare quality, reflecting a general optimism regarding its benefits. However, a notable gap in training was identified, with 86% of respondents reporting no prior instruction in telemedicine. This finding underscores the need for integrating telemedicine education into medical training programs, a recommendation supported by previous studies. For instance, the introduction of telemedicine training modules at a German medical university demonstrated significant improvements in students' knowledge, skills and attitudes toward telemedicine [15]. Similarly, research from Australia highlighted that medical students with practical exposure to telemedicine were more likely to correctly define e‐health and telemedicine, further emphasizing the value of incorporating hands‐on telemedicine experiences into educational curricula [16]. Moreover, a training program conducted for staff nurses in Bengaluru demonstrated that it is an effective approach to raise awareness about telemedicine and promote its adoption [17]. The overwhelming support (84.4%) for such initiatives reflects students' recognition of its relevance, though some skepticism may arise from concerns about already demanding academic schedules.

Interestingly, while students overwhelmingly supported the inclusion of telemedicine training, they favored its introduction as an elective course. This preference stems from their perception of the heavy workload in medical education, as highlighted in open‐ended responses. The literature on this topic discusses both optional and mandatory training programs [18], with their associated advantages and disadvantages. Integrating mandatory telemedicine courses into the curriculum has the potential to significantly enhance students' perceptions and attitudes toward this field. Evidence from an Australian study revealed a statistically significant improvement in students' views following their participation in telemedicine workshops, highlighting the transformative impact of structured educational interventions [19]. Other recommendations suggest that such courses should be conducted in 2–3 sessions, each lasting no more than 1 h [20, 21].

Regarding the role of telemedicine in optimizing medical activities, 51.5% of respondents expressed a willingness to adopt telemedicine in their future practice. These results align with the general trend described in previous studies [16, 22]. However, 39% of medical students adopted a neutral position. This neutrality likely reflects uncertainty regarding the practical applications of telemedicine and its limitations across different clinical contexts, particularly in the absence of sufficient practical exposure. In our study, familiarity and prior use were significantly associated with positive perceptions, indicating that increased exposure could enhance acceptance.

An important finding of this study is the substantial proportion of neutral responses observed across multiple attitudinal items, ranging up to 46.4%. Higher levels of neutrality were identified for complex or potentially controversial aspects of telemedicine, such as malpractice risk, its role in current medical practice, and the conditions under which it should be used.

This pattern suggests that a considerable proportion of students may not yet have well‐defined opinions on these aspects, likely reflecting limited practical exposure and insufficient integration of telemedicine into formal medical training.

Furthermore, subgroup analyses showed consistently higher proportions of neutral responses among students who were not familiar with telemedicine, reinforcing the association between exposure and the ability to form clear attitudes. These findings highlight the presence of an intermediate group of students characterized by uncertainty rather than clear acceptance or rejection, which may represent a key target for educational interventions.

Respondents highlighted several advantages of telemedicine, including time efficiency, continuous monitoring for chronic conditions, improved access to specialists and saving costs, which are already documented in the literature [11, 22, 23, 24, 25, 26]. These benefits align with telemedicine's potential to address systemic inefficiencies in Romanian healthcare, particularly in underserved areas. However, challenges such as the limitations of remote physical examinations, preference for in‐person interactions, technical issues and concerns about data security temper this enthusiasm [23, 24, 27]. These concerns resonate with broader global debates on telemedicine's limitations and the need for robust infrastructure.

The specific utility of telemedicine for chronic care, routine consultations and family medicine was widely recognized, both in our study and in previous research [28, 29], suggesting its potential to address gaps in these areas. However, skepticism about its role in emergencies (491 respondents disagreeing) reflects valid concerns about its appropriateness for critical care. These findings underscore the importance of delineating the contexts in which telemedicine can be most effectively utilized.

Statistical analyses further illuminated key relationships. Younger students, were more familiar with telemedicine, suggesting that generational differences might influence adaptability to new technologies. Interestingly, gender did not significantly impact perceptions or usage, emphasizing a generally uniform attitude across genders in this cohort. Academic year and prior telemedicine use were predictive of positive perceptions, suggesting that increased exposure and clinical experience enhance acceptance.

Multivariable regression analyses provided further insight into the factors independently associated with telemedicine‐related outcomes. Familiarity with telemedicine was significantly associated with both academic progression and familiarity with recent medical innovations, suggesting that exposure to medical knowledge and innovation plays a central role in shaping awareness.

Perceptions of telemedicine as having a positive impact on healthcare quality were independently associated only with familiarity with medical innovations, indicating that attitudes toward innovation may be a stronger determinant than demographic or educational factors.

In addition, familiarity with medical innovations and prior telemedicine training were significant predictors of perceiving telemedicine as contributing to the optimization of medical activities. Willingness to adopt telemedicine in future practice was associated with both gender and familiarity with innovations, with female students showing higher willingness.

Overall, these findings emphasize the importance of exposure to innovation and structured training as key drivers of telemedicine acceptance, beyond traditional demographic characteristics.

The qualitative feedback provided by students highlighted actionable recommendations, such as improving internet access in rural areas, raising public awareness and addressing technological and data security challenges. These insights underscore the systemic and infrastructural changes required for telemedicine to reach its full potential.

In conclusion, the findings of this study reveal an optimistic yet nuanced view of telemedicine among Romanian medical students. While they recognize its potential to enhance healthcare, they also acknowledge its limitations and the need for structured training and systemic improvements. Addressing these challenges will be critical to ensuring that telemedicine becomes an integral part of future medical practice.

### 4.1 Study Limitations and Future Research Directions

This study has several limitations that warrant consideration. First, the cross‐sectional design captures perceptions at a single point in time, limiting the ability to assess changes in attitudes or familiarity with telemedicine as students progress through their medical education. Second, the reliance on self‐reported data introduces potential biases, including social desirability bias, which may have influenced responses about the perceived benefits and utility of telemedicine.

Moreover, the use of convenience sampling may have introduced selection bias, as participation was voluntary and may have attracted students with a greater interest in digital health or innovation. Therefore, the findings should be interpreted with caution and may not be fully representative of the entire population of Romanian medical students.

Additionally, although the sample included students from all major medical university centers in Romania, it was predominantly composed of female respondents. This imbalance may reflect the gender distribution typically observed in medical schools, where female students are often overrepresented, but it may also introduce a degree of response bias. Previous research suggests that gender differences may influence attitudes toward digital health technologies, including telemedicine, with some studies indicating higher levels of acceptance and engagement among female participants. Consequently, the predominance of female respondents in this study may have contributed to the generally positive perceptions observed and may limit the generalizability of the findings to the entire population of medical students.

Another limitation lies in the unequal representation of academic years, with higher participation from first‐ and fourth‐year students, which may skew the results toward the perspectives of these groups. Finally, the study did not explore in depth external factors, such as institutional policies or regional healthcare disparities, which could further influence students' perceptions of telemedicine.

Future research could address these limitations by employing a longitudinal design to track changes in telemedicine perceptions over time, particularly as students gain more clinical experience. Additionally, qualitative research methods, such as interviews or focus groups, could yield richer insights into students' concerns, preferences and perceived barriers to telemedicine adoption. Exploring the impact of targeted telemedicine training programs on knowledge, skills and attitudes would also be valuable, as would investigating the integration of telemedicine into specific clinical specialties.

Lastly, comparative studies between different countries or regions could highlight cultural and systemic factors influencing telemedicine adoption and inform strategies for its effective implementation in diverse healthcare settings.

## 5 Conclusions

This study sheds light on the complex attitudes of Romanian medical students towards telemedicine, revealing both optimism and caution about its integration into healthcare practices. The findings indicate a general consensus on the positive impact of telemedicine in enhancing access to care, particularly for chronic disease management and routine consultations, while highlighting significant reservations about its application in emergency care and its ability to replace traditional consultations.

A key finding is the significant gap in formal telemedicine training within medical curricula, despite most students expressing interest in such programs. This emphasizes the need for academic institutions to incorporate telemedicine education to prepare future healthcare professionals for its integration.

Challenges such as poor internet connectivity, low public awareness and data security concerns also emerged as barriers. Addressing these issues through targeted initiatives and investments will be crucial for building trust and enabling widespread adoption.

In conclusion, while telemedicine holds great promise in improving healthcare accessibility and quality, its successful implementation requires a multifaceted approach. This includes enhancing medical education, addressing infrastructural challenges and fostering public trust. By bridging these gaps, the Romanian healthcare system can empower future medical professionals to integrate telemedicine into their practices, ultimately advancing the efficiency and inclusivity of healthcare delivery.

## Author Contributions

**Andreea‐Ramona Treteanu:** conceptualization, investigation, writing – original draft, data curation, formal analysis, methodology, visualization. **Ștefan Busnatu:** conceptualization, investigation, writing – review and editing, methodology, supervision, formal analysis. **Liviu‐Ionuț Șerbănoiu:** conceptualization, investigation, writing – original draft, methodology, formal analysis, data curation. **Iulian Năstasă:** conceptualization, investigation, writing – review and editing, validation, formal analysis, supervision. **Octavian Andronic:** supervision, data curation, formal analysis, project administration, writing – review and editing, methodology, conceptualization.

## Funding

The authors have nothing to report.

## Ethics Statement

The work being submitted has been done in accordance with Wiley's Best Practice Guidelines on Publishing Ethics, in an ethical and responsible way, with no research misconduct, which includes, but is not limited to data fabrication and falsification, plagiarism, image manipulation, unethical research, biased reporting, authorship abuse, redundant or duplicate publication, and undeclared conflicts of interest.

## Conflicts of Interest

There are no conflicts of interest.

## STROBE Statement

This study was reported in accordance with the Strengthening the Reporting of Observational Studies in Epidemiology (STROBE) statement for cross‐sectional studies. A completed STROBE checklist is provided as supporting material.

## Transparency Statement

The manuscript is an honest, accurate, and transparent account of the study being reported; that no important aspects of the study have been omitted; and that any discrepancies from the study as planned (and, if relevant, registered) have been explained.

## Supporting information

Supporting File

## Data Availability

The data that support the findings of this study are available from the corresponding author upon reasonable request.
