# Supplementary material for: Attitude, Awareness and Knowledge of Telemedicine Among Romanian Medical Students: A Cross‐Sectional Study
Source: Health Sci Rep. 2026 Jun 22;9(6):e72695. doi: 10.1002/hsr2.72695 (PMC13284888; doi:10.1002/hsr2.72695)
Supplement: Supplementary file 1 — Supporting File [file HSR2-9-e72695-s001.pdf]

STROBE Statement—Checklist of items that should be included in reports of ***cross-sectional studies***

	Item No	Recommendation	Location in manuscript
**Title and abstract**	1	(*a*) Indicate the study's design with a commonly used term in the title or the abstract	Title and Abstract
		(*b*) Provide in the abstract an informative and balanced summary of what was done and what was found	Abstract
**Introduction**			
Background/rationale	2	Explain the scientific background and rationale for the investigation being reported	Introduction
Objectives	3	State specific objectives, including any prespecified hypotheses	End of introduction
**Methods**			
Study design	4	Present key elements of study design early in the paper	Materials and Methods – Study design
Setting	5	Describe the setting, locations, and relevant dates, including periods of recruitment, exposure, follow-up, and data collection	Materials and Methods – Study setting and period
Participants	6	(*a*) Give the eligibility criteria, and the sources and methods of selection of participants	Materials and Methods – Study population
Variables	7	Clearly define all outcomes, exposures, predictors, potential confounders, and effect modifiers. Give diagnostic criteria, if applicable	Materials and Methods – Questionnaire design
Data sources/ measurement	8*	For each variable of interest, give sources of data and details of methods of assessment (measurement). Describe comparability of assessment methods if there is more than one group	Materials and Methods – Questionnaire + Data collection
Bias	9	Describe any efforts to address potential sources of bias	Materials and Methods – Sampling + Limitations section
Study size	10	Explain how the study size was arrived at	Materials and Methods – Sample size consideration
Quantitative variables	11	Explain how quantitative variables were handled in the analyses. If applicable, describe which groupings were chosen and why	Materials and Methods – Statistical analysis
Statistical methods	12	(*a*) Describe all statistical methods, including those used to control for confounding	Materials and Methods – Statistical analysis
		(*b*) Describe any methods used to examine subgroups and interactions	Materials and Methods – Statistical analysis
		(*c*) Explain how missing data were addressed	Materials and Methods – Statistical analysis
		(*d*) If applicable, describe analytical methods taking account of sampling strategy	Materials and Methods – Statistical analysis
		(*e*) Describe any sensitivity analyses	Materials and Methods – Statistical analysis

**Results**			
Participants	13*	(a) Report numbers of individuals at each stage of study—eg numbers potentially eligible, examined for eligibility, confirmed eligible, included in the study, completing follow-up, and analysed	Materials and Methods – Data collection procedure
		(b) Give reasons for non-participation at each stage	Materials and Methods – Data collection
		(c) Consider use of a flow diagram	Not included (optional)
Descriptive data	14*	(a) Give characteristics of study participants (eg demographic, clinical, social) and information on exposures and potential confounders	Results – Tables 2 & 3
		(b) Indicate number of participants with missing data for each variable of interest	Materials and Methods – exclusion criteria
Outcome data	15*	Report numbers of outcome events or summary measures	Results section
Main results	16	(*a*) Give unadjusted estimates and, if applicable, confounder-adjusted estimates and their precision (eg, 95% confidence interval). Make clear which confounders were adjusted for and why they were included	Results – regression tables
		(*b*) Report category boundaries when continuous variables were categorized	Results – Likert scale tables
		(*c*) If relevant, consider translating estimates of relative risk into absolute risk for a meaningful time period	Not applicable
Other analyses	17	Report other analyses done—eg analyses of subgroups and interactions, and sensitivity analyses	Results – subgroup + regression
**Discussion**			
Key results	18	Summarise key results with reference to study objectives	Discussions
Limitations	19	Discuss limitations of the study, taking into account sources of potential bias or imprecision. Discuss both direction and magnitude of any potential bias	Limitations section
Interpretation	20	Give a cautious overall interpretation of results considering objectives, limitations, multiplicity of analyses, results from similar studies, and other relevant evidence	Discussions section
Generalisability	21	Discuss the generalisability (external validity) of the study results	Limitations section
**Other information**			
Funding	22	Give the source of funding and the role of the funders for the present study and, if applicable, for the original study on which the present article is based	Funding section

*Give information separately for exposed and unexposed groups.

Note: An Explanation and Elaboration article discusses each checklist item and gives methodological background and published examples of transparent reporting. The STROBE checklist is best used in conjunction with this article (freely available on the Web sites of PLoS Medicine at http://www.plosmedicine.org/, Annals of Internal Medicine at http://www.annals.org/, and Epidemiology at http://www.epidem.com/). Information on the STROBE Initiative is available at www.strobe-statement.org.
